# Hemorrhagic Shock: Blood Marker Sequencing and Pulmonary Gas Exchange

**DOI:** 10.3390/diagnostics13040639

**Published:** 2023-02-09

**Authors:** Benedikt Treml, Axel Kleinsasser, Johann Knotzer, Robert Breitkopf, Corinna Velik-Salchner, Sasa Rajsic

**Affiliations:** 1 Department of Anesthesia and Intensive Care Medicine, Medical University Innsbruck, 6020 Innsbruck, Austria; 2 Klinikum Wels-Grieskirchen, 4600 Wels, Austria

**Keywords:** shock, hemorrhage, pulmonary gas exchange, hyperglycemia, lactate, base excess

## Abstract

Background: The early identification of internal hemorrhage in critically ill patients may be difficult. Besides circulatory parameters, hemoglobin and lactate concentration, metabolic acidosis and hyperglycemia serve as laboratory markers for bleeding. In this experiment, we examined pulmonary gas exchange in a porcine model of hemorrhagic shock. Moreover, we sought to investigate if a chronological order of appearance regarding hemoglobin, lactatemia, standard base excess/deficit (SBED) and hyperglycemia exists in early severe hemorrhage. Methods: In this prospective, laboratory study, twelve anesthetized pigs were randomly allocated to exsanguination or a control group. Animals in the exsanguination group (*n* = 6) endured a 65% blood loss over 20 min. No intravenous fluids were administered. Measurements were taken before, immediately after, and at 60 min after the completed exsanguination. Measurements included pulmonary and systemic hemodynamic variables, hemoglobin concentration, lactate, base excess (SBED), glucose concentration, arterial blood gases, and a multiple inert gas assessment of pulmonary function. Results: At baseline, variables were comparable. Immediately after exsanguination, lactate and blood glucose were increased (*p* = 0.001). The arterial partial pressure of oxygen was increased at 60 min after exsanguination (*p* = 0.04) owing to a decrease in intrapulmonary right-to-left shunt and less ventilation-perfusion inequality. SBED was different to the control only at 60 min post bleeding (*p* < 0.001). Hemoglobin concentration did not change at any time (*p* = 0.97 and *p* = 0.14). Conclusions: In experimental shock, markers of blood loss became positive in chronological order: lactate and blood glucose concentrations were raised immediately after blood loss, while changes in SBED lagged behind and became significant one hour later. Pulmonary gas exchange is improved in shock.

## 1. Introduction

Acute internal hemorrhage is a medical emergency often encountered in critical care units. Acute bleeding following surgery may not be overt when no drainage tubes were inserted, or such tubes are obstructed. Clinical signs such as changes in blood pressure or tachycardia may be masked by medication such as catecholamines or beta-blockade. This is where modern blood gas analyzers may be used, as some of the parameters examined may also serve as markers of acute bleeding. 

Hemoglobin concentration is widely used as a biomarker for blood loss but also known to lag behind. The drop in hemoglobin concentration depends on how fast interstitial fluid is pulled from the tissues and on the amount of fluids administered [1]. Furthermore, lactatemia may indicate hypoperfusion. When oxygen delivery is critically low, cells will partially switch from the Krebs cycle to anaerobic glycolysis, resulting in more pyruvate/lactate. Lactate promotes the release of hydrogen ions, resulting in acidemia. This connection between lactatemia and acidemia was shown more than 50 years ago [2]. A correlation between lactate and prognosis was later displayed in a review article by MacLean and others [3]. 

In shock, epinephrine triggers glycogenolysis and when more pyruvate is produced than consumed by the Krebs cycle, lactate increases [4,5]. Lactate levels rise rapidly, for instance in elite master swimmers, lactate levels as high as 14 mmol/L (127 mg/dL) are reached shortly after 50 m of front crawl [6]. 

Plasma base concentrations are carefully monitored in critical care medicine and deflections have been used to gauge transfusion requirements on admission to the emergency room [7]. Today, mostly standard base excess/deficit (SBED) is used. SBED reflects the amount of virtual bicarbonate necessary to elevate the pH to 7.40 at a fixed arterial partial pressure of carbon dioxide (aPCO2) of 40 mmHg. This eliminates the respiratory leg of this parameter and SEBD reflects metabolic acidosis alone. Relevant base deficits are regularly found in the bleeding patient [7]. 

Hyperglycemia is an indicator of stress of every description. In 1878, Claude Bernard presented a notion of the reaction of blood sugar to hemorrhage [8], later termed “stress hyperglycemia” since it reflects a response to stress, illness and the like. Stress hyperglycemia is thought to be protective, allowing for survival in periods of strain. 

Finally, the question arises if severe hemorrhagic shock affects pulmonary blood flow and consequently the balance of lung ventilation and perfusion. We thus applied the multiple inert gas elimination technique to assess pulmonary ventilation/perfusion distribution. Moreover, we sought to elucidate which of the above markers indicate massive hemorrhage first, and if there is a temporal sequence. We hypothesized that lactatemia was the first biomarker to indicate acute hemorrhage, since lactate levels can rise instantly, as known from sports physiology [6].

## 2. Materials and Methods

### 2.1. Overview

**Figure 1:**
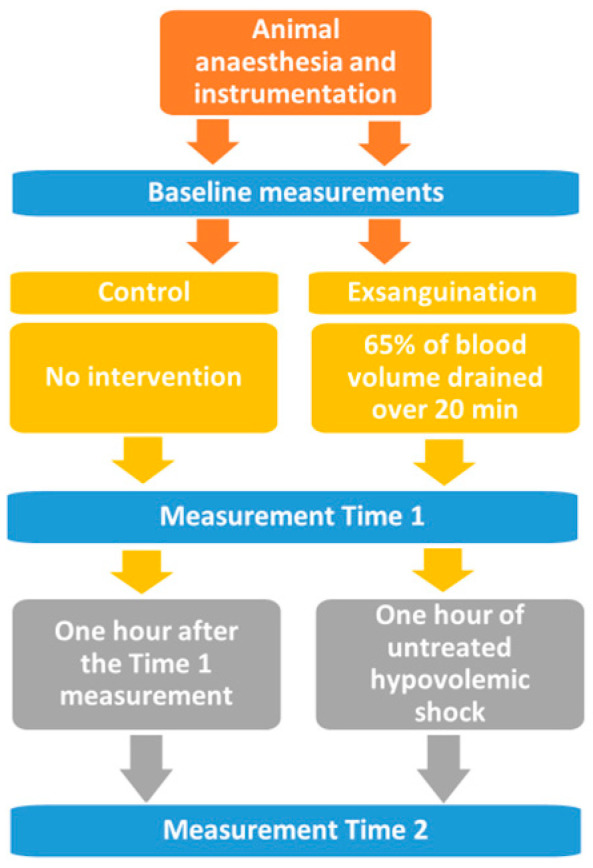
Summary of the study design and the timing of measurements taken. Measurement Time 1: Immediately after 65% blood loss in the experimental group, and simultaneous blood sampling in the control group; Measurement Time 2: One hour after 65% blood loss in the experimental group, and simultaneous blood sampling in the control group.

Given the principles of the 3Rs (Replacement, Reduction and Refinement) in animal research, we chose the lowest sample size which we deemed acceptable from a statistical point of view [9]. In this controlled laboratory study, we aimed to form a severe hemorrhagic shock in anesthetized pigs. Twelve pigs were randomly allocated to exsanguination or a control group. Animals in the exsanguination group (*n* = 6) endured a 65% blood loss over 20 min, while the animals in the control group received only anaesthesia and regular blood sampling, as per protocol for both groups. No intravenous fluids were administered, and no other interventions were carried out. In a series of four pilot experiments, we found that 65% blood loss resulted in a severe shock which the pigs survived for a little longer than one hour. Therefore, we aimed for one hour since we wanted to examine both acute and protracted hemorrhagic shock. A sketch of the study timeline is given in Figure 1. 

### 2.2. Animal Anesthesia and Instrumentation

All experiments conformed to the guidelines of the US National Institutes of Health, the guide for the care and use of laboratory animals and were approved by the Austrian Federal Animal Investigational Committee. Twelve healthy, 12-week-old crossbreed pigs of either gender were selected from a local stock with veterinary care. Anesthesia was induced in the test facility, with ketamine (25 mg/kg intramuscularly) and atropine (0.01 mg/kg intravenously). After tracheal intubation, propofol was started at a rate of 10 mg/kg/h. Lungs were mechanically ventilated in volume-controlled mode (Evita-2; Draeger, Luebeck, Germany) with a fraction of inspired oxygen of 0.21 and a positive end-expiratory pressure of 5 cm H_2_O. Tidal volumes were adjusted to 7 to 10 mL/kg at 15 breaths per minute (fixed rate) to achieve an arterial partial pressure of carbon dioxide (PaCO_2_) between 35 mmHg and 40 mmHg. Anesthesia was maintained using continuous propofol and boluses of an opioid drug (piritramide, 15 mg per bolus, equipotent to 10 mg morphine) when the paw test indicated insufficient depth of anesthesia. Throughout the experimental runs, no fluids were administered. A standard lead II electrocardiogram was used to monitor cardiac rhythm. Body temperature was measured using the indwelling thermistor-tipped Swan-Ganz catheter and maintained between 38.5 °C and 39.0 °C (physiologic body temperature of the pig) by using a heating blanket. An 8.5F catheter was advanced from the internal jugular vein into a branch of the pulmonary artery to measure central venous pressure and mean pulmonary arterial pressure (mPAP). Cardiac output was assessed using the thermodilution technique (10 mL cooled saline in triplicates) after taking the blood samples. A 6.0F arterial catheter introduced into the femoral artery was used to monitor systemic blood pressure and to take blood samples. This catheter was also used for exsanguination over a period of 20 min, assuming that blood volume equaled 7% of body weight. Catheters were filled with saline and connected to pressure transducers zeroed to ambient pressure at the level of the right atrium. Measurements included hemodynamic, ventilatory, blood gas and inert gas parameters. Arterial blood and multiple inert gas samples were taken at baseline, immediately after drawing 65% blood volume (Time 1), and at 60 min after drawing 65% blood volume (Time 2). The blood sampling was simultaneously performed in the control group. Ventilator settings were not changed during the experimental period. All experiments complied with relevant legislation to ensure animal welfare. All laboratory and hemodynamic parameters are provided in the Tables S1–S3.

This study was approved by the Austrian Federal Animal Investigational Committee (BMWF-66.011/0066-II/10b/2009).

### 2.3. Blood Gas Analyses, Lactate, Glucose, SBED and Hemoglobin

A Radiometer ABL800 FLEX (Radiometer, Krefeld, Germany) blood gas analyzer was used for arterial blood gas analyses and the determination of lactate, glucose, SBED and hemoglobin. The average haemoglobin concentration in piglets ranges between 10.5 ± 2.2 g/dL and 12.4 ± 2.0 [9,10].

### 2.4. Multiple Inert Gas Elimination Technique-MIGET

The multiple inert gas elimination technique [11,12] helps to determine the distributions of ventilation (VA) and perfusion (Q) in the lung. With the MIGET, 6 gases of different blood-gas solubility are intravenously infused. Then, how the lung excretes or retains these gases in the blood is measured. Sulfurhexafluoride is a gas with poor blood gas solubility and is used to identify shunting lung units, the lung units with blood flow but no ventilation. The other gases used have a higher blood gas solubility (ethane > cyclopropane > isoflurane > diethyl ether), helping to define low, normal, and high VA/Q lung units. Acetone is the most soluble gas and helps to determine dead space. The residual sum of squares (RSS) is used as an indicator of fit of the data to the model [11,12].

### 2.5. Statistical Evaluation

Statistical analyses were performed using SPSS (Version 22.0. Released 2013, IBM Corp., Armonk, NY, USA) and Microsoft Excel (Microsoft Corporation, Redmond, WD, USA, Version 2212). To test the normality of data, we used the Shapiro–Wilk test. An analysis of variance was used to determine intergroup and intragroup differences. Significant results were post-hoc analyzed using the Newman–Keuls test to eliminate false-positive observations (alpha error). Results are given as the mean with standard deviation (SD). A significance level of 0.05 was applied.

## 3. Results

### 3.1. Lactate, Glucose, SBED and Hemoglobin Concentration

Lactate, glucose, SBED and hemoglobin concentration are displayed in Table 1. Lactate (*p* = 0.001) and glucose (*p* = 0.002) became different to control immediately after blood loss, while SBED was depressed only at the 60-min post measurement (*p* = 0.001). Regarding hemoglobin concentration, no difference between the exsanguination and the control group could be seen at any time point (Table 1). 

The analysis of the results within the same group, but at the different times (baseline, time 1 and time 2) is presented in Table S4. We found significant difference in the trend of all parameters, except for haemoglobin in the haemorrhage group of animals. Within the control group, none of the parameters changed significantly over the time. 

### 3.2. Hemodynamic Measurements

At baseline, heart rate, pulmonary and systemic blood pressure were comparable between groups (Table 2). Heart rate (*p* = 0.001) was raised and blood pressure was depressed after 65% blood loss (systolic arterial pressure, central venous pressure, mean pulmonary arterial pressure and pulmonary arterial occlusion pressure *p* < 0.001). 

The analysis of the parameters within the same group but at the different times (baseline, time 1 and time 2) is presented in Table S4. We found a significant difference in the trend of all parameters. Within the control group, none of the parameters changed significantly over the time.

### 3.3. Blood Gas Analyses and Multiple Inert Gas Elimination Technique: Pulmonary Gas Exchange

Blood gas analyses and pulmonary gas exchange variables are given in Table 1 and Table 3. The arterial partial pressure of oxygen improved after exsanguination. In shock, pulmonary right-to-left shunt was virtually gone and the remaining pulmonary perfusion became more homogenous (Table 3).

Arterial partial pressure of oxygen was improved 60 min after hemorrhage, due to both a reduction in intrapulmonary right-to-left shunt and a reduction in LogSDQ after 20 min. LogSDQ stands for log standard deviation of the width of the perfusion distribution and reflects the extent of ventilation-perfusion inequality (i.e., smaller LogSDQ would present lower ventilation-perfusion inequality) [11,12]. Moreover, with low cardiac output, pulmonary transit times are prolonged, accordingly no diffusion limitation could be detected. Quality of the inert gas analyses as indicated by RSS showed that all the RSS were below 10.6, and 91% were below 5.3, indicating good data quality.

The analysis of the parameters within the same group, but at the different times (baseline, time 1 and time 2) is presented in Table S4. We found significant difference in the trend of all parameters, except in low VA/Q and the normal VA/Q. Within the control group, none of the parameters changed significantly over the time.

## 4. Discussion

In this animal study on laboratory findings in the first hour after major bleeding, we observed that only lactate and blood sugar increased immediately after hemorrhage, while base deficit lagged behind and hemoglobin did not change at all. When working with critically ill patients, hemorrhage sometimes only becomes evident when blood loss is substantial enough to alter hemodynamics. Then, typically, a blood sample is run through the blood gas analyzer to see if hemoglobin has changed. Hemoglobin, however, is not the early train.

### 4.1. Hemoglobin and Hemorrhage

A drop in hemoglobin did not occur in our study despite a reasonably long observational period. This was due to the experimental design of this study as we did not administer any fluid, as this would have brought in extrinsic factors such as chloride from saline or lactate from lactated Hartmann’s. Fluids are the single most important first intervention in shock but known to dilute the remaining blood. Thus, the drop in hemoglobin concentration depended on how fast interstitial fluid was pulled from the tissues. This fluid shift was slow and the difference in hemoglobin concentration remained insignificant compared to the control at all measured time points. Thus, hemoglobin alone may not be authentic for hemorrhage. However, when fluids are administered, as in the emergency department, early hemoglobin changes can be used as a marker of blood loss [13].

### 4.2. Pulmonary Gas Exchange in Shock

Evidence on pulmonary gas exchange in the setting of hemorrhagic shock is still scarce. In a previous experiment, pulmonary vasodilation together with an increase in cardiac output has been shown to worsen pulmonary gas exchange [14]. In the setting of increased cardiac output, pulmonary blood flow was redistributed to lung units with subnormal ventilation-perfusion ratios (low VA/Q or right-to-left shunt) [14]. 

The opposite seems to occur in a severe hemorrhagic shock. In the present study, pulmonary blood flow was severely decreased but ventilation was left unchanged. In this setting, we observed decreased pulmonary right-to-left shunt and an increase in arterial partial pressure of oxygen (Table 3). In addition, LogSDQ reflecting the extent of ventilation-perfusion inequality became smaller at the 60-min measurement, which is compatible with an improvement of arterial oxygenation. Thus, in hemorrhagic shock, pulmonary gas exchange is enhanced, and the intergroup difference of 15 mmHg in arterial partial pressure of oxygen is remarkably high. 

### 4.3. Low Cardiac Output and Intrapulmonary Right-to-Left Shunt

It is well established that there is a positive correlation between increases in shunt and cardiac output [14,15,16]. Our data provide evidence that a fall in cardiac output also causes a fall in pulmonary shunt. As an explanation, Lynch and coworkers suggested that shunt and non-shunt blood vessels behaved differently when challenged with a change in cardiac output [16]. While we cannot corroborate or reject this notion, vascular de-recruitment seems plausible to the authors.

In summary, pulmonary gas exchange in severe hypovolemia turns out to be more efficient than during normovolemia. Of course, it cannot be deduced from this experiment whether this is an evolutionary, protective mechanism, but improved pulmonary gas exchange clearly is advantageous in the setting of hypovolemic shock.

### 4.4. Lactatemia

Lactatemia was an early indicator of hemorrhage and shock in this experiment. The origin of lactatemia in shock is still not entirely elucidated. Lactatemia frequently reflects perfusion deficits of skeletal muscles and is not necessarily a sign of poor vital organ perfusion [17]. However, it may also be a result of epinephrine release [18], which likely is the case in our study. Our results go well with those of Moomey and coworkers, who examined the prognostic value of lactate and SBED in an animal model of hemorrhagic shock [19]. In Moomey’s study, only lactate but not SBED was related to blood loss, leading to a conclusion that the lactate is rather characteristic for hemorrhage.

However, lactate may be raised in the patients with a vitamin B1 deficiency, as the thiamine-dependent pyruvate dehydrogenase presents the link between cytosolic glycolysis and the citric acid cycle. In case of thiamine deficiency, lactate prevails over pyruvate and patients with malnutrition may have significantly higher lactate levels. There are many causes for increased lactic acid including hyperglycemia, metformin use, diabetes, mitochondrial dysfunction and more. Accordingly, lactate alone cannot be a sensitive marker for acute hemorrhage. 

### 4.5. Standard Base Deficit (SBED)

In this experiment, SBED was measured at minus seven at the 60-min measurement. Standard base deficit has been used in emergency medicine for some time, and in the experiment of Moomey and coworkers, SBED was determined by the magnitude of injury [19].

It has to be noted that in our experiment, SBED reacted instantaneously to hemorrhage, but variance at the second time point (immediately after 65% blood loss) was too large to grant significance (Table 1). Standard base deficit was not associated with systolic blood pressure in a study using the National Trauma Data Base of the American college of Surgeons, so SEBD may not stand as the laboratory representative of shock [20]. Nonetheless, SEBD seemed to be a sensitive indicator in our study. Standard base deficit was shown to be a predictor of death in other investigations [21]. 

The biology behind the decrease in SBED in hemorrhage is thought to be a defect in oxygen utilization and a reflection of the plasma lactate level [22]. This concept goes well with our results, where first the lactate is increased, followed with the SBED drop (Table 1).

### 4.6. Hyperglycemia

Stress hyperglycemia is considered to provide fuel in times of need. Hypovolemia causes release of catecholamines [23] and increased plasma catecholamines mediate glycolysis in the liver and muscle [24]. In our experiment, hyperglycemia was an instant response directly after hemorrhage. 

While epinephrine is one cause of hyperglycemia, also blood sugar raising cortisol is released in stress from the adrenal gland. Cortisol increases blood sugar through gluconeogenesis (liver) and glycogenolysis in the liver and muscle. Cortisol is known to have a sustained effect on blood sugar, and offsets the action of insulin on fat cells. Moreover, glucocorticoids may even cause diabetes [25]. 

### 4.7. Relationships and Dependencies of the Observed Parameters

There are certain interactions between the parameters observed which need to be considered. Lactate is a product of anaerobic glycolysis, and both hyperglycemia and lactatemia are a result of raised epinephrine. Standard base deficit is calculated from arterial pH and paCO_2_, but arterial pH in turn may depend on the concentration of lactate, which in turn is raised by epinephrine. Epinephrine thus affects blood sugar, lactic acid, arterial pH and base deficit. Epinephrine by itself could be a sensitive biomarker. However, plasma catecholamines are difficult to measure. Given that increases in blood sugar may cause lactatemia, base deficit and acidosis, hyperglycemia seems the most independent of the observed parameters.

### 4.8. Prehospital Trauma Care: On-Site Hyperglycemia Screening for Hemorrhage?

Rises in blood sugar immediately after bleeding might be used for on-site screening for major hemorrhage and helping to decide whether fast transport (airborne) is mandatory or ground transport will suffice. In the prehospital setting, a commercially available blood glucose meter may help to detect hyperglycemia. Such glucose meters are already on-board in many ambulances.

Admission to the emergency department presents a similar situation, where hyperglycemia might help to identify those requiring immediate care.

### 4.9. Limitations

It is a shortcoming of this study that epinephrine, norepinephrine and cortisol have not been measured. This would have provided information on whether hypovolemia, stress, or both, caused hyperglycemia.

## 5. Conclusions

In shock, prompt care is essential. Our findings may contribute to the early diagnosis of internal hemorrhage after cardiac or aortic surgery or any other clandestine bleeding (i.e., gastrointestinal hemorrhage). Pulmonary gas exchange in shock is improved owing to a reduction in intrapulmonary right-to-left shunt. Shunt regularly appears in anesthetized animals, thus in spontaneously breathing subjects, this effect may be less pronounced. 

Lactatemia and hyperglycemia, together with heart rate and blood pressure depression, may serve as indicators of hemorrhage with a very recent onset. Substantially negative SBED indicate longer-standing bleeding. 

## Figures and Tables

**Table 1:** Early and late laboratory indicators in acute hemorrhagic shock.

Laboratory Parameter	Baseline	*p* Value	Time 1 ^a^	*p* Value	Time 2 ^a^	*p* Value
**Lactate (** **mg/dL)**						
Hemorrhage	15 ± 2	0.984	44 ± 3	0.001	95 ± 24	<0.001
Control	15 ± 3		14 ± 2		15 ± 2	
**Glucose (** **mg/dL)**						
Hemorrhage	95 ± 15	0.941	138 ± 35	0.002	161 ± 37	<0.001
Control	95 ± 12		97 ± 15		93 ± 11	
**SBED (mmol/l)**						
Hemorrhage	3.4 ± 2.9	0.649	−0.1 ± 1.9	0.270	−7.2 ± 3.6	0.001
Control	4.2 ± 2.6		2.7 ± 3.4		2.7 ± 2.3	
**Hemoglobin (** **g/dL)**						
Hemorrhage	7.1 ± 0.7	0.864	7.2 ± 0.5	0.973	6.3 ± 1.1	0.143
Control	7.1 ± 0.8		7.2 ± 0.9		7.5 ± 0.7	
**apH**						
Hemorrhage	7.48 ± 0.04	0.828	7.47 ± 0.04	0.846	7.30 ± 0.09	0.001
Control	7.49 ± 0.05		7.47 ± 0.05		7.44 ± 0.04	

^a^ Time 1: Immediately after 65% blood loss in the experimental group, and simultaneous blood sampling in the control group; Time 2: One hour after 65% blood loss in the experimental group, and simultaneous blood sampling in the control group. Abbreviations: SBED: standard base excess or deficit, reflects standard base excess or deficit in mmol/L; apH: arterial pH. Hemorrhage reflects animals which were 65% exsanguinated and control animals in the control group (no blood loss).

**Table 2:** Circulatory parameters in acute hemorrhagic shock.

Circulatory Parameters	Baseline	*p* Value	Time 1 ^a^	*p* Value	Time 2 ^a^	*p* Value
**Heart Rate (beats/min)**						
Hemorrhage	89 ± 11	0.976	151 ± 39	0.001	164 ± 51	<0.001
Control	84 ± 12		85 ± 14		84 ± 11	
**Systolic arterial pressure (mmHg)**						
Hemorrhage	105 ± 15	0.840	47 ± 3	<0.001	50 ± 5	<0.001
Control	107 ± 15		114 ± 16		104 ± 15	
**CVP (mmHg)**						
Hemorrhage	9 ± 2	0.983	−3 ± 6	<0.001	0 ± 1	<0.001
Control	9 ± 2		9 ± 2		28 ± 3	
**PAPmean (mmHg)**						
Hemorrhage	26 ± 4	0.994	8 ± 5	<0.001	13 ± 2	<0.001
Control	26 ± 4		24 ± 2		28 ± 3	
**PCWP (mmHg)**						
Hemorrhage	11 ± 3	0.958	3 ± 2	0.001	2 ± 1	<0.001
Control	10 ± 2		11 ± 2		11 ± 3	
**Cardiac Output (liters/minute)**						
Hemorrhage	5.1 ± 0.6	0.822	2.6 ± 0.8	<0.001	2.0 ± 0.3	<0.001
Control	5.2 ± 0.8		5.7 ± 0.9		5.5 ± 0.8	

^a^ Time 1: Immediately after 65% blood loss in the experimental group, and simultaneous blood sampling in the control group; Time 2: One hour after 65% blood loss in the experimental group, and simultaneous blood sampling in the control group. Abbreviations: CVP: central venous pressure; PAPmean: mean pulmonary arterial pressure; PCWP: pulmonary arterial occlusion pressure. Hemorrhage reflects animals, which were 65% exsanguinated and control animals in the control group (no blood loss).

**Table 3:** Pulmonary gas exchange in acute hemorrhagic shock.

Laboratory Parameters	Baseline	*p* Value	Time 1 ^a^	*p* Value	Time 2 ^a^	*p* Value
**PaO_2_ (mmHg)**						
Hemorrhage	74 ± 8	0.903	80 ± 12	0.492	90 ± 6	0.042
Control	73 ± 8		72 ± 6		78 ± 5	
**PaCO_2_ (mmHg)**						
Hemorrhage	37 ± 2	0.974	32 ± 3	0.038	38 ± 3	0.993
Control	37 ± 2		37 ± 2		38 ± 2	
**SVO_2_ (%)**						
Hemorrhage	60 ± 14	0.746	28 ± 15	0.005	24 ± 7	0.002
Control	58 ± 12		58 ± 13		60 ± 13	
**Shunt (% of cardiac output)**						
Hemorrhage	3.9 ± 1.7	0.473	1.3 ± 0.8	0.042	0.7 ± 0.4	0.021
Control	3.2 ± 1.7		4.0 ± 1.8		4.1 ± 1.5	
**Low VA/Q (%)**						
Hemorrhage	0 ± 0	1.000	0 ± 0	1.000	0 ± 0	1.000
Control	0 ± 0		0 ± 0		0 ± 0	
**Normal VA/Q (%)**						
Hemorrhage	96 ± 2	0.470	98 ± 1	0.332	99 ± 0	0.020
Control	97 ± 2		96 ± 2		96 ± 2	
**Mean of Q**						
Hemorrhage	1.07 ± 0.21	0.839	2.61 ± 0.84	<0.001	2.66 ± 0.55	<0.001
Control	0.91 ± 0.23		0.95 ± 0.29		0.90 ± 0.33	
**LogSDQ**						
Hemorrhage	0.46 ± 0.11	0.843	0.70 ± 0.18	0.045	0.40 ± 0.07	0.727
Control	0.48 ± 0.14		0.46 ± 0.12		0.50 ± 0.14	

^a^ Time 1: immediately after 65% blood loss in the experimental group, and simultaneous blood sampling in the control group; Time 2: one hour after 65% blood loss in the experimental group, and simultaneous blood sampling in the control group. Abbreviations: PaO_2_: arterial partial pressure of oxygen; PaCO_2_: arterial partial pressure of carbon dioxide; SVO_2_: mixed venous oxygen saturation; Normal VA/Q: perfusion of lung units with a normal ventilation perfusion ratio; Low VA/Q: blood flow to lung units with a low ventilation perfusion ratio; LogSDQ: log standard deviation of the perfusion distribution; Mean of Q; the mean of the perfusion distribution; Shunt: the blood flow to unventilated lung units. Hemorrhage reflects animals, which were 65% exsanguinated and control animals in the control group (no blood loss).

## Data Availability

The datasets used and analyzed during the current study are made available from the corresponding author on reasonable request.

## Supplementary Materials

The following supporting information can be downloaded at: https://www.mdpi.com/article/10.3390/diagnostics13040639/s1, Table S1. Laboratory parameters of experimental animals (arterial sample, *n* = 12), Table S2. Laboratory parameters of experimental animals (venous sample, *n* = 12), Table S3. Hemodynamic parameters of experimental animals (*n* = 12), Table S4. Analysis of laboratory and hemodynamic parameters within the same animal group (control *n* = 6, haemorrhage *n* = 6).
