# Supplementary material for: Hemorrhagic Shock: Blood Marker Sequencing and Pulmonary Gas Exchange
Source: Diagnostics (Basel). 2023 Feb 9;13(4):639. doi: 10.3390/diagnostics13040639 (PMC9955920; doi:10.3390/diagnostics13040639)
Supplement: Supplementary file 1 [file diagnostics-13-00639-s001.zip › diagnostics-2155139-supplementary.pdf]

diagnostics

Article

Hemorrhagic Shock: Blood Marker Sequencing and Pulmonary Gas Exchange

Benedikt Treml, Axel Kleinsasser, Johann Knotzer, Robert Breitkopf, Corinna Velik-Salchner and Sasa Rajsic

Supplementary material

Contents

Table S1. Laboratory parameters of experimental animals (arterial sample, n = 12)

Table S2. Laboratory parameters of experimental animals (venous sample, n = 12)

Table S3. Hemodynamic parameters of experimental animals (n = 12)

Table S4. Analysis of laboratory and hemodynamic parameters within the same animal group (control n = 6, haemorrhage n = 6)

Table S1. Laboratory parameters of experimental animals (arterial sample, n = 12)

Control animal group			
**Animal 1**	**Baseline**	**Time 1**	**Time 2**
Hb	6	6.8	6.7
Hkt	18	20	20
paO2	65.6	80.1	74.7
paCO2	34.1	33.9	39.1
pHa	7.562	7.449	7.479
BE	7.8	-0.9	4.5
HCO3	30	23	28.4
ctO2		9.3	9
sO2	95.3	96.6	95.6
NA	145	151.3	142.9
K	4.09	3.29	3.65
Lactate	14	15.1	19.5
AnGap	16.1	25.6	12.2
Glucose	119	111	91
**Animal 2**			
Hb	6.1	8.7	7.7
Hkt	18	26	23
paO2	72.9	79	75.2
paCO2	35.7	40.3	39.9
pHa	7.517	7.39	7.395
BE	5	-1	-0.9
HCO3	28.3	23.8	23.9
ctO2	8.3	11.6	10.6
sO2	96.3	94.5	93.3
NA	140.5	144.2	141
K	3.51	3.35	4
Lactate	10.7	14.6	17
AnGap	10.7	13.7	18.1
Glucose	84	99	99
**Animal 3**			
Hb	8.1	7.5	7.7
Hkt	24	22	23
paO2	63.8	71.6	88.2
paCO2	37.5	39.4	35.9
pHa	7.496	7.416	7.496
BE	4.8	0.2	3.7
HCO3	28.3	24.8	27.1
ctO2	10.5	9.9	10.7
sO2	92.8	94	98.2

NA	142.3	143.5	146.2
K	3.88	3.52	3.29
Lactate	14.9	11.8	13.9
AnGap	10.9	9.3	13.4
Glucose	91	77	83
**Animal 4**			
Hb	7.7	6	6.8
Hkt	23	18	20
paO2	88.2	65.6	80.1
paCO2	35.9	34.1	33.9
pHa	7.496	7.562	7.449
BE	3.7	7.8	-0.9
HCO3	27.1	30	23
ctO2	10.7	X	9.3
sO2	98.2	95.3	96.6
NA	146.2	145	151.3
K	3.29	4.09	3.29
Lactate	13.9	14	15.1
AnGap	13.4	16.1	25.6
Glucose	83	119	111
**Animal 5**			
Hb	6.7	6.1	8.7
Hkt	20	18	26
paO2	74.7	72.9	79
paCO2	39.1	35.7	40.3
pHa	7.479	7.517	7.39
BE	4.5	5	-1
HCO3	28.4	28.3	23.8
ctO2	9	8.3	11.6
sO2	95.6	96.3	94.5
NA	142.9	140.5	144.2
K	3.65	3.51	3.35
Lactate	19.5	10.7	14.6
AnGap	12.2	10.7	13.7
Glucose	91	84	99
**Animal 6**			
Hb	7.7	8.1	7.5
Hkt	23	24	22
paO2	75.2	63.8	71.6
paCO2	39.9	37.5	39.4
pHa	7.395	7.496	7.416
BE	-0.9	4.8	0.2

HCO3	23.9	28.3	24.8
ctO2	10.6	10.5	9.9
sO2	93.3	92.8	94
NA	141	142.3	143.5
K	4	3.88	3.52
Lactate	17	14.9	11.8
AnGap	18.1	10.9	9.3
Glucose	99	91	77
**Haemorrhage animal group**			
**Animal 1**	**Baseline**	**Time 1**	**Time 2**
Hb	7.5	6.5	5.4
Hkt	22	19	16
paO2	71.6	88.5	83.8
paCO2	39.4	31.3	40.5
pHa	7.416	7.477	7.238
BE	0.2	-0.8	-9.6
HCO3	24.8	22.6	16.9
ctO2	9.9	9	7.2
sO2	94	97.7	93
NA	143.5	142.1	142.7
K	3.52	4.1	3.81
Lactate	11.8	24.5	106
AnGap	9.3	9.6	16.6
Glucose	77	89	182
**Animal 2**			
Hb	6	7.5	5
Hkt	18	22	15
paO2	65.6	62.1	83.6
paCO2	34.1	34.1	39.7
pHa	7.562	7.495	7.218
BE	7.8	2.4	-10.9
HCO3	30	25.7	15.8
ctO2	X	9.6	6.6
sO2	95.3	91.8	92.4
NA	145	144.3	142.1
K	4.09	5.09	3.82
Lactate	14	48.7	118.1
AnGap	16.1	20.7	19.1
Glucose	119	199	203
**Animal 3**			
Hb	6.8	6.7	5.1
Hkt	20	20	19

paO2	80.1	79.3	83.9
paCO2	33.9	31	42.8
pHa	7.449	7.434	7.177
BE	-0.9	-3.5	-11.7
HCO3	23	20.3	15.5
ctO2	9.3	9.1	6.1
sO2	96.6	95.9	92.7
NA	151.3	146	141.5
K	3.29	4.13	3.74
Lactate	15.1	62.3	128.2
AnGap	25.6	19.8	17.7
Glucose	111	155	207
**Animal 4**			
Hb	7.7	8.1	7
Hkt	23	24	21
paO2	88.2	96	94.4
paCO2	35.9	28.7	36.8
pHa	7.496	7.537	7.38
BE	3.7	1.4	-3.5
HCO3	27.1	23.8	21.3
ctO2	10.7	11	9.8
sO2	98.2	98.5	98.7
NA	146.2	143.4	142.4
K	3.29	3.73	3.16
Lactate	13.9	31	72.5
AnGap	13.4	14.3	14.3
Glucose	83	129	131
**Animal 5**			
Hb	6.7	7	7.4
Hkt	20	21	22
paO2	74.7	67	97.7
paCO2	39.1	39.7	34.4
pHa	7.479	7.403	7.403
BE	4.5	-0.5	-3.4
HCO3	28.4	24.2	21
ctO2	9	8.7	10.3
sO2	95.6	91.4	98.6
NA	142.9	142	142.3
K	3.65	3.94	3.22
Lactate	19.5	48.5	73.1
AnGap	12.2	16.7	14.5
Glucose	91	145	126

**Animal 6**			
Hb	8.1	7.3	7.7
Hkt	24	21	23
paO2	63.8	87.7	93.6
paCO2	37.5	28.3	34.2
pHa	7.496	7.526	7.394
BE	4.8	0.3	-4
HCO3	28.3	22.9	20.4
ctO2	10.5	9.7	10.7
sO2	92.8	97.8	99.3
NA	142.3	142.4	143.5
K	3.88	4.93	3.32
Lactate	14.9	47.8	71.1
AnGap	10.9	16.4	18.4
Glucose	91	108	118

Abbreviations: SBED: standard Base Excess or Deficit, reflects standard base excess or deficit in mmol/L; apH: arterial pH. Hemorrhage reflects animals, which were 65% exsanguinated and control animals in the control group (no blood loss). Time 1: Immediately after 65% blood loss in the experimental group, and simultaneous blood sampling in the control group; Time 2: One hour after 65% blood loss in the experimental group, and simultaneous blood sampling in the control group.

Table S2. Laboratory parameters of experimental animals (venous sample, n = 12)

Control animal group			
**Animal 1**	**Baseline**	**Time 1**	**Time 2**
pvO2	27.7	39.4	33.1
pvCO2	44.1	41.3	47.3
pHv	7.505	7.395	7.403
Be v	10	-0.1	3.7
HCO3 v	34	24.7	28.8
ctO2 v	3.5	5.9	9.2
sO2 v	34.3	62.2	33.1
NA v	140.8	145.9	142.1
K v	4.67	3.63	3.71
Lactatee v	13.8	18.3	21.1
AnGap v	10.5	20.8	11
Glucose v	128	119	96
**Animal 2**			
pvO2	33.2	47	38.5
pvCO2	42.6	44.3	43.1
pHv	7.467	7.385	7.366
Be v	5.9	0.7	-1.2
HCO3 v	30.1	25.9	24.1
ctO2 v	4.7	9	6
sO2 v	55.6	74.2	58.5
NA v	137.5	140.6	138.2
K v	3.67	3.62	4.16
Lactate v	11.1	14.7	14.4
AnGap v	8.1	9.3	16.2
Glucose v	88	104	102
**Animal 3**			
pvO2	33.5	43.4	37.9
pvCO2	44.2	45.9	42
pHv	7.44	7.38	7.443
Be v	4.7	1.2	3.6
HCO3 v	29.3	26.5	28.1
ctO2 v	6.1	7.2	6.8
sO2 v	55.1	67.3	63.6
NA v	139	140	143.9
K v	4.02	3.81	3.43
Lactate v	18.1	13.2	14.1
AnGap v	7.7	6.3	11.3
Glucose v	94	84	89
**Animal 4**			

pvO2	37.9	27.7	39.4
pvCO2	42	44.1	41.3
pHv	7.443	7.505	7.395
Be v	3.6	10	-0.1
HCO3 v	28.1	34	24.7
ctO2 v	6.8	3.5	5.9
sO2 v	63.6	34.3	62.2
NA v	143.9	140.8	145.9
K v	3.43	4.67	3.63
Lactate v	14.1	13.8	18.3
AnGap v	11.3	10.5	20.8
Glucose v	89	128	119
**Animal 5**			
pvO2	33.1	33.2	47
pvCO2	47.3	42.6	44.3
pHv	7.403	7.467	7.385
Be v	3.7	5.9	0.7
HCO3 v	28.8	30.1	25.9
ctO2 v	9.2	4.7	9
sO2 v	33.1	55.6	74.2
NA v	142.1	137.5	140.6
K v	3.71	3.8	3.62
Lactate v	21.1	11.1	14.7
AnGap v	11	8.1	9.3
Glucose v	96	88	104
**Animal 6**			
pvO2	38.5	33.5	43.4
pvCO2	43.1	44.2	45.9
pHv	7.366	7.44	7.38
Be v	-1.2	4.7	1.2
HCO3 v	24.1	29.3	26.5
ctO2 v	6	6.1	7.2
sO2 v	58.5	55.1	67.3
NA v	138.2	139	140
K v	4.16	4.02	3.81
Lactate v	14.4	18.1	13.2
AnGap v	16.2	7.7	6.3
Glucose v	102	94	84
**Haemorrhage animal group**			
**Animal 1**	**Baseline**	**Time 1**	**Time 2**
pvO2	43.4	21.4	21.7
pvCO2	45.9	43.7	70.6

pHv	7.38	7.356	7.032
Be v	1.2	-1.5	-11.2
HCO3 v	26.5	23.9	18.3
ctO2 v	7.2	3.5	1
sO2 v	67.3	42.8	13.4
NA v	140	141	139.4
K v	3.81	3.71	4.02
Lactate v	13.2	15.2	108.6
AnGap v	6.3	3.8	15.1
Glucose v	84	69	190
**Animal 2**			
pvO2	27.7	16.7	25
pvCO2	44.1	53.6	71.6
pHv	7.505	7.352	7.018
Be v	10	2.9	-11.7
HCO3 v	34	29.1	18
ctO2 v	3.5	1.5	1.6
sO2 v	34.3	12.4	21.3
NA v	140.8	142.7	139.5
K v	4.67	5.48	3.97
Lactate v	13.8	20	122.8
AnGap v	10.5	18.1	14.5
Glucose v	128	214	207
**Animal 3**			
pvO2	39.4	35.1	24.3
pvCO2	41.3	43.5	71.3
pHv	7.395	7.374	7.008
Be v	-0.1	-0.4	-12.2
HCO3 v	24.7	24.8	17.5
ctO2 v	5.9	4.9	1.3
sO2 v	62.2	52.3	17.3
NA v	145.9	141.6	139.2
K v	3.63	4.39	3.93
Lactate v	18.3	54.8	128.5
AnGap v	20.8	13.2	15.6
Glucose v	119	125	217
**Animal 4**			
pvO2	37.9	25.7	29.1
pvCO2	42	48.4	51
pHv	7.443	7.349	7.285
Be v	3.6	0.2	-3.1
HCO3 v	28.1	26.1	23.7

ctO2 v	6.8	3.5	4.2
sO2 v	63.6	30.7	31.9
NA v	143.9	141.4	140.2
K v	3.43	3.87	3.37
Lactate v	14.1	32	73.2
AnGap v	11.3	11.2	11.9
Glucose v	89	135	136
**Animal 5**			
pvO2	33.1	20.5	28.6
pvCO2	47.3	54.8	52.6
pHv	7.403	7.307	7.28
Be v	3.7	0.3	-2.7
HCO3 v	28.8	26.8	24.2
ctO2 v	3.2	1.8	3.6
sO2 v	33.1	17.5	30.6
NA v	142.1	140.5	139.3
K v	3.71	3.97	3.42
Lactate v	21.1	46	74.6
AnGap v	11	12.7	10.6
Glucose v	96	144	137
**Animal 6**			
pvO2	33.5	13.9	27.8
pvCO2	44.2	56.6	52.5
pHv	7.44	7.283	7.278
Be v	4.7	-0.7	-2.8
HCO3 v	29.3	26.2	24
ctO2 v	6.1	1.2	3.4
sO2 v	55.1	11.9	28.6
NA v	139	139.2	140.4
K v	4.02	5.52	3.49
Lactate v	18.1	53.2	71.8
AnGap v	7.7	13.5	13.9
Glucose v	94	125	126

Abbreviations: v: venous sample; SBED: standard Base Excess or Deficit, reflects standard base excess or deficit in mmol/L; apH: arterial pH. Hemorrhage reflects animals, which were 65% exsanguinated and control animals in the control group (no blood loss). Time 1: Immediately after 65% blood loss in the experimental group, and simultaneous blood sampling in the control group; Time 2: One hour after 65% blood loss in the experimental group, and simultaneous blood sampling in the control group.

Table S3. Hemodynamic parameters of experimental animals (n = 12)

Control animal group			
**Animal 1**	**Baseline**	**Time 1**	**Time 2**
HZV	5	5.00	5.40
VE(STPD)	8.1	6.7	7.8
VE(BTPS)	10.5	8.7	10.1
VE(ATPS)	9.6	7.9	9.2
HF	69	98	92
SYS	100	88	109
DIA	54	50	58
CVP	5	11	11
PPAm	21	26	33
PCWP	9	14	12
etCO2	35	34	39
PEAK	40.0	35.0	36.0
MEAN	11	11	8
f	18	18	18
P50	36.0	32.0	36.0
**Animal 2**			
HZV	6.90	6.6	5.10
VE(STPD)	6.7	5.5	5.8
VE(BTPS)	8.7	7.1	7.6
VE(ATPS)	7.9	6.5	6.9
HF	80	67	73
SYS	119	130	92
DIA	79	103	60
CVP	11	7	8
PPAm	22	25	28
PCWP	11	9	12
etCO2	38	37	36
PEAK	32.0	31.0	36
MEAN	8	7	7
f	18	18	18
P50	32.0	32.0	32.0
**Animal 3**			
HZV	4.40	6.4	4.40
VE(STPD)	6.0	6	5.6
VE(BTPS)	8.1	8	7.6
VE(ATPS)	7.4	7.3	6.9
HF	104	90	83
SYS	133	112	90
DIA	88	75	50

CVP	11	10	7
PPAm	24	25	28
PCWP	12	11	6
etCO2	34	37	35
PEAK	41.0	35.0	38.0
MEAN	10	10	8
f	18	18	18
P50	33.0	32.0	33.0
**Animal 4**			
HZV	4.40	5	5.00
VE(STPD)	5.6	8.1	6.7
VE(BTPS)	7.6	10.5	8.7
VE(ATPS)	6.9	9.6	7.9
HF	83	69	98
SYS	90	100	88
DIA	50	54	50
CVP	7	5	11
PPAm	28	21	26
PCWP	6	9	14
etCO2	35	35	34
PEAK	38.0	40.0	35.0
MEAN	8	11	11
f	18	18	18
P50	33.0	36.0	32.0
**Animal 5**			
HZV	5.40	6.90	6.6
VE(STPD)	7.8	6.7	5.5
VE(BTPS)	10.1	8.7	7.1
VE(ATPS)	9.2	7.9	6.5
HF	92	80	67
SYS	109	119	130
DIA	58	79	103
CVP	11	11	7
PPAm	33	22	25
PCWP	12	11	9
etCO2	39	38	37
PEAK	36.0	32.0	31.0
MEAN	8	8	7
f	18	18	18
P50	36.0	32.0	32.0
**Animal 6**			
HZV	5.10	4.40	6.4

VE(STPD)	5.8	6.0	6
VE(BTPS)	7.6	8.1	8
VE(ATPS)	6.9	7.4	7.3
HF	73	104	90
SYS	92	133	112
DIA	60	88	75
CVP	8	11	10
PPAm	28	24	25
PCWP	12	12	11
etCO2	36	34	37
PEAK	36	41.0	35.0
MEAN	7	10	10
f	18	18	18
P50	32.0	33.0	32.0
**Haemorrhage animal group**			
**Animal 1**	**Baseline**	**Time 1**	**Time 2**
HZV	6.4	2.2	2.00
VE(STPD)	6	6.7	6.7
VE(BTPS)	8	9.0	9.0
VE(ATPS)	7.3	8.2	8.2
HF	90	129	114
SYS	112	50	44
DIA	75	28	13
CVP	10	-3	2
PPAm	25	7	13
PCWP	11	4	2
etCO2	37	32	27
PEAK	35.0	35.0	37.0
MEAN	10	10	8
f	18	18	18
P50	32.0	32.0	32.0
**Animal 2**			
HZV	5	2	2.00
VE(STPD)	8.1	10.3	6.7
VE(BTPS)	10.5	13.4	9.0
VE(ATPS)	9.6	12.3	8.2
HF	69	129	110
SYS	100	50	45
DIA	54	30	15
CVP	5	-14	0
PPAm	21	-3	16
PCWP	9	X	0

etCO2	35	32	29
PEAK	40.0	40.0	37.0
MEAN	11	11	8
f	18	18	18
P50	36.0	36.0	32.0
**Animal 3**			
HZV	5.00	2.00	1.30
VE(STPD)	6.7	10.3	6.7
VE(BTPS)	8.7	13.4	9.0
VE(ATPS)	7.9	12.3	8.2
HF	98	104	115
SYS	88	47	50
DIA	50	26	15
CVP	11	6	0
PPAm	26	11	17
PCWP	14	X	1
etCO2	34	35	32
PEAK	35.0	35.0	37.0
MEAN	11	11	8
f	18	18	18
P50	32.0	32.0	32.0
**Animal 4**			
HZV	4.40	2.70	2.00
VE(STPD)	5.6	8.2	8.2
VE(BTPS)	7.6	11.0	11.0
VE(ATPS)	6.9	10.0	10.0
HF	83	181	215
SYS	90	40	60
DIA	50	20	20
CVP	7	0	0
PPAm	28	8	11
PCWP	6	0	2
etCO2	35	29	29
PEAK	38.0	38.0	38.0
MEAN	8	8	8
f	18	18	18
P50	33.0	33.0	33.0
**Animal 5**			
HZV	5.40	4.30	2.30
VE(STPD)	7.8	10.3	8.2
VE(BTPS)	10.1	13.4	11.0
VE(ATPS)	9.2	12.25	10.0

HF	92	223	213
SYS	109	48	50
DIA	58	32	30
CVP	11	-5	0
PPAm	33	13	11
PCWP	12	6	2
etCO2	39	44	29
PEAK	36.0	36.0	39.0
MEAN	8	8	9
f	18	18	18
P50	36.0	36.0	33.0
**Animal 6**			
HZV	4.40	2.20	2.30
VE(STPD)	6.0	10.3	8.2
VE(BTPS)	8.1	13.4	11.0
VE(ATPS)	7.4	12.25	10.0
HF	104	142	215
SYS	133	45	50
DIA	88	26	25
CVP	11	-4	0
PPAm	24	10	11
PCWP	12	2	2
etCO2	34	28	29
PEAK	41.0	41.0	38.0
MEAN	10	10	8
f	18	18	18
P50	33.0	33.0	33.0

Abbreviations: CVP: central venous pressure; PAPmean: mean pulmonary arterial pressure; PCWP: pulmonary arterial occlusion pressure; PaO_2: arterial partial pressure of oxygen; $PaCO_2$: arterial partial pressure of carbon dioxide; SVO_2: mixed venous oxygen saturation; Normal VA/Q: perfusion of lung units with a normal ventilation perfusion ratio; Low VA/Q: blood flow to lung units with a low ventilation perfusion ratio; LogSDQ: log standard deviation of the perfusion distribution; Mean of Q; the mean of the perfusion distribution; Shunt: the blood flow to unventilated lung units. Hemorrhage reflects animals, which were 65% exsanguinated and control animals in the control group (no blood loss). Time 1: Immediately after 65% blood loss in the experimental group, and simultaneous blood sampling in the control group; Time 2: One hour after 65% blood loss in the experimental group, and simultaneous blood sampling in the control group.

Table S4. Analysis of laboratory and hemodynamic parameters within the same animal group (control n = 6, haemorrhage n = 6)

Laboratory parameter	Baseline	Time 1	P value (Baseline/Time1)	Time 2	P value (Time1/Time2)
**Lactate (mg/dL)**					
Hemorrhage	15 ±2	44 ±3	0.001	95 ±24	<0.001
Control	15 ±3	14 ±2	0.972	15 ±2	0.962
**Glucose (mg/dL)**					
Hemorrhage	95 ±15	138 ±35	0.003	161 ±37	<0.001
Control	95 ±12	97 ±15	0.976	93 ±11	0.918
**SBED (mmol/l)**					
Hemorrhage	3.4 ±2.9	-0.1 ±1.9	0.220	-7.2 ±3.6	<0.001
Control	4.2 ±2.6	2.7 ±3.4	0.667	2.7 ±2.3	0.277
**Hemoglobin (g/dL)**					
Hemorrhage	7.1 ±0.7	7.2 ±0.5	0.918	6.3 ±1.1	0.194
Control	7.1 ±0.8	7.2 ±0.9	0.989	7.5 ±0.7	0.865
**apH**					
Hemorrhage	7.48 ±0.04	7.47 ±0.04	0.904	7.30 ±0.09	0.001
Control	7.49 ±0.05	7.47 ±0.05	0.948	7.44 ±0.04	0.573
**Circulatory parameters**					
**Heart Rate (beats/min)**					
Hemorrhage	89 ±11	151 ±39	<0.001	164 ±51	<0.001
Control	84 ±12	85 ±14	0.996	84 ±11	0.982
**Systolic arterial pressure (mmHg)**					
Hemorrhage	105 ±15	47 ±3	<0.001	50 ±5	<0.001
Control	107 ±15	114 ±16	0.421	104 ±15	0.903
**CVP (mmHg)**					
Hemorrhage	9 ±2	-3 ±6	<0.001	0 ±1	0.001
Control	9 ±2	9 ±2	0.998	28 ±3	0.930
**PAPmean (mmHg)**					
Hemorrhage	26 ±4	8 ±5	<0.001	13 ±2	<0.001
Control	26 ±4	24 ±2	0.365	28 ±3	0.799
**PCWP (mmHg)**					
Hemorrhage	11 ±3	3 ±2	0.001	2 ±1	0.001
Control	10 ±2	11 ±2	0.882	11 ±3	0.814
**Cardiac Output (liters/minute)**					
Hemorrhage	5.1 ±0.6	2.6 ±0.8	<0.001	2.0 ±0.3	<0.001
Control	5.2 ±0.8	5.7 ±0.9	0.481	5.5 ±0.8	0.527
**Pulmonary gas exchange**					
**PaO2 (mmHg)**					
Hemorrhage	74±8	80±12	0.434	90±6	0.022
Control	73±8	72±6	0.802	78±5	0.600
**PaCO2 (mmHg)**					
Hemorrhage	37±2	32±3	0.019	38±3	0.847
Control	37±2	37±2	0.902	38±2	0.820
**SVO2 (%)**					
Hemorrhage	60±14	28±15	0.013	24±7	0.008
Control	58±12	58±13	0.564	60±13	0.642
**Shunt (% of cardiac output)**					
Hemorrhage	3.9±1.7	1.3±0.8	0.033	0.7±0.4	0.016

Control	3.2±1.7	4.0±1.8	0.654	4.1±1.5	0.784
**Low VA/Q (%)**					
Hemorrhage	0±0	0±0	1.000	0±0	1.000
Control	0±0	0±0	1.000	0±0	1.000
**Normal VA/Q (%)**					
Hemorrhage	96±2	98±1	0.875	99±0	0.943
Control	97±2	96±2	0.853	96±2	0.988
**Mean of Q**					
Hemorrhage	1.07±0.21	2.61±0.84	<0.001	2.66±0.55	<0.001
Control	0.91±0.23	0.95±0.29	0.884	0.90±0.33	0.972
**LogSDQ**					
Hemorrhage	0.46±0.11	0.70±0.18	0.040	0.40±0.07	0.790
Control	0.48±0.14	0.46±0.12	0.974	90±6	0.705

Time 1: Immediately after 65% blood loss in the experimental group, and simultaneous blood sampling in the control group; Time 2: One hour after 65% blood loss in the experimental group, and simultaneous blood sampling in the control group. Abbreviations: SBED: standard Base Excess or Deficit, reflects standard base excess or deficit in mmol/L; apH: arterial pH; CVP: central venous pressure; PAPmean: mean pulmonary arterial pressure; PCWP: pulmonary arterial occlusion pressure; PaO2: arterial partial pressure of oxygen; PaCO2: arterial partial pressure of carbon dioxide; SVO2: mixed venous oxygen saturation; Normal VA/Q: perfusion of lung units with a normal ventilation perfusion ratio; Low VA/Q: blood flow to lung units with a low ventilation perfusion ratio; LogSDQ: log standard deviation of the perfusion distribution; Mean of Q; the mean of the perfusion distribution; Shunt: the blood flow to unventilated lung units. Haemorrhage reflects animals, which were 65% exsanguinated and control animals in the control group (no blood loss).
